# SARS-CoV-2 Viral Shedding and Associated Factors among COVID-19 Inpatients and Outpatients

**DOI:** 10.1155/2022/1411106

**Published:** 2022-06-12

**Authors:** Masoomeh Sofian, Behzad Khansarinejad, Ehsanollah Ghaznavi-Rad, Farzaneh Shokoohi, Hossein Mazaherpour, Farzane Farmani, Mona Sadat Larijani, Leila Pakpour, Amitis Ramezani

**Affiliations:** ^1^Infectious Disease Research Center (IDRC), Arak University of Medical Science, Arak, Iran; ^2^Department of Microbiology and Immunology, Arak University of Medical Sciences, Arak, Iran; ^3^Department of Infectious Disease, Arak University of Medical Science, Arak, Iran; ^4^School of Medicine, Arak University of Medical Sciences, Arak, Iran; ^5^Clinical Research Dept., Pasteur Institute of Iran, Tehran, Iran

## Abstract

**Background:**

According to the contagious ability of the new virus, SARS-CoV-2, characterization of viral shedding duration in the period of infection is highly valuable in terms of providing quarantine guidelines and isolation policies. Therefore, we aimed at viral shedding determination in 58 COVID-19 confirmed Iranian subjects in different stages.

**Methods:**

58 COVID-19 confirmed Iranian subjects including 21 outpatients and 37 inpatients were investigated. The analytical data and clinical properties were documented in the standard questionnaire. The RT-PCR tests were done two and three weeks after the symptoms initiation.

**Results:**

Viral eradication occurred in 44.8% two weeks after illness initiation whereas in 71% who achieved a negative PCR test in the third week. Moreover, prolonged viral shedding was observed in hospitalized cases in comparison to outpatients. Almost 30% of patients continued viral shedding three weeks after disease initiation.

**Conclusion:**

A longer duration of viral shedding in hospitalized cases rather than outpatients was observed in this study. The results similar to other investigations call into question if the current policies are enough to prevent the viral spread or not. This study should be done on a larger sample to provide an appropriate time in isolation policy.

## 1. Introduction

SARS-CoV-2 as the cause of COVID-19 infection has affected global health since December, 2019. Most COVID-19 infected individuals show mild clinical symptoms whereas the small group who develop severe and complicated faces of the virus [1, 2]. The infection could occur in all ages and spread easily; however, the likelihood of developing a severe clinical course of the infection may increase with different medical status like cardiovascular diseases, diabetes, chronic respiratory diseases, and hypertension [2–4]. Several studies have investigated to picture COVID-19 clinical features and also specific antibody response to SARS-CoV-2 [5–8]. The viral transmission is supposed to happen mainly via droplet spread and close contact although the airborne transmission is specifically in the form of aerosols which is the new concern. Several vaccine platforms are in development besides the approved approaches. However, the reduction of viral transmission in the community is still essential [9, 10]. In addition, the contagiousness of the virus, specific antibody reactions, virus shedding kinetics, and the time sequences of clinical manifestations must be more discussed to make the virus pathophysiology clear and provide better infection control strategies [11–13].

Molecular approaches are more suitable for accurate diagnosis of specific pathogens. The successful PCR technique is associated with the virus genomic and possible changes in protein expression due to possible mutations which might affect the PCR procedure [14, 15]. Reverse-transcription real-time PCR is the preferable procedure for COVID-19 diagnosis. The detection of viral load is dependent on the time after illness onset. During the first two weeks after onset, this virus could reliably be detected mostly in sputum followed by nasal swabs, whereas throat swabs were found to be unreliable 8 days after symptom onset [16, 17]. High viral shedding during the first week of symptoms was observed in a study with a peak on day 4 [18]. The viral shedding duration assessment is among the current challenges, especially for recovered patients. Duration of viral shedding after recovery from the symptoms is not still exactly clear. The viral shedding duration is normally taken into attention for a precise isolation policy and it also plays an infectivity marker role [9, 15, 19].

The researching data have shown that sustained viral shedding is associated with severe presentation of the disease [20, 21]. Previously, MERS-CoV prolonged virus shedding was reported in animal models of immunosuppression [22]. In a study by Hail et al. on MERS-CoV, diabetes was found to be associated with prolonged detection of respiratory MERS-CoV RNA [23]. Recently, Liu et al. have reported a COVID-19 subject whose viral shedding prolonged for forty-six days with some chronic infections as his underlying diseases [24]. Another study also demonstrated that patients having high temperature at the admission time had longer SARS-CoV-2 shedding [25]. Therefore, according to unknown features of the virus, it seems necessary to determine the viral shedding period in different populations to control the viral transmission by a standard discharge protocol.

In this study, SARS-CoV-2 viral shedding among the infected cases was explored. Moreover, the correlated factors with longer COVID-19 disease were assessed.

## 2. Materials and Methods

Totally, 58 individuals with COVID-19 symptoms who were admitted to Imam Reza clinic and Ayatollah Khansari Hospital, Arak, Iran were enrolled from March 2020 to May 2020. Written informed consent was obtained from all subjects upon investigation according to the Declaration of Helsinki, and the study was approved by the ethics committee of Arak University of Medical Sciences (IR.ARAKMU.REC.1399.002).

The standard protocol for COVID-19 diagnosis was followed according to the provided flowchart in Iran [26]. The swab specimens were collected and viral RNA was isolated (QIAamp DSP Virus Kit Qiagen, Hilden, Germany) according to the provided protocol. The RT-PCR tests (Sansure biotech, Changsha, China) were then done in accordance to the instruction.

Analytical data and clinical outcomes were recorded in the designed questionnaire. COVID-19 confirmed cases were evaluated for viral shedding two and three weeks after illness onset. Moreover, RT-PCR test results, resolved respiratory symptoms, normal temperature, and improved lesions on chest computed tomography (CT) images were considered for hospital discharge. It should be noted that patients were admitted to the ICU due to the lungs involvement based on clinical and radiological evidence (CT scans).

The chi-square test was applied in the SPSS 16 package program in addition to Pearson's correlation coefficient test. *P* value <0.05 was reported significant.

## 3. Results

**Figure 1:**
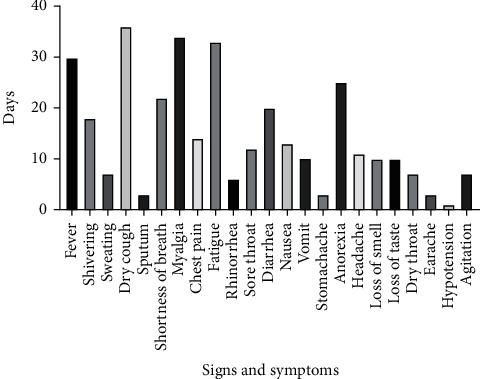
Frequency of COVID-19 patients signs and symptoms.

This study was conducted on fifty-eight COVID-19 subjects (30 males and 28 females) to determine the viral shedding duration and associated factors. The demographic data is shown in Table 1. The studied population included 37 hospitalized and 21 outpatients. The mean age was 49.36 years (21 to 87). The most prevalent symptoms were dry cough, myalgia, and fatigue, respectively (Figure 1).

Thirty-one percent of investigated cases had underlying diseases and diabetes mellitus was the most common one (6.9%). Twenty-seven subjects had an exposure history to an infected person, and 14 patients had an infected individual in their first-grade family.

Among the 89.7% of the studied subjects who had a chest CT scan, 75.9% showed abnormality (Table 1). The most common involvement was ground-glass opacity. Furthermore, among all 58 patients, 5 (9%) were monitored in the ICU, 32 (55%) patients were admitted to the infectious ward, and 21 (36%) were outpatients. The duration of time in the ICU varied in those 5 patients ranging from 2 to 35 days.

55 of the cases (94.8%) were treated by antibiotics (mostly Levofloxacin for 5 days) and 21 of them got Chloroquine alone. None of the ICU patients received chloroquine alone, in fact they took combination therapy including chloroquine. One of the five patients admitted in the ICU, expired. The demographic data and patients' outcomes are presented in Table 1. The exposure time and the first patient's symptoms were variable from four to fifteen days.

The clearance of the virus was achieved after two weeks in 44.8% of the subjects and also in 71% of the cases that happened beyond the third week. Overall, 29% of patients continued viral shedding after three RT-PCR tests. Moreover, comparison of 21 outpatients and 37 hospitalized cases indicated that the second PCR became negative in nonhospitalized patients earlier than the hospitalized ones and viral shedding was longer in severe forms of the infection (*P* value = 0.04).

There was no correlation between the applied medicine or therapeutic regimen and the second/third PCR positivity (*P*value >0.05). Furthermore, the second PCR positive result correlation with some factors including ICU presence, WBC, liver function tests, platelet count, LDH, CRP, CPK, sex, and age was investigated and it showed that it was only associated with low hemoglobin level (less than 12 g/dl) (*P* value = 0.018).

There was also a correlation between gender and ICU/ventilation supply indicating that males were more in need of these supplies (*P* value = 0.024). In other words, the admitted cases to ICU were just men. The applied antibiotics and therapeutic regimen were not associated with the second/third PCR positivity results (*P* value = 0.84, *P* value = 0.118)

## 4. Discussion

The confirmed patients were investigated to determine the viral shedding prolongation by RT-PCR test two/three times. The clearance of the virus was achieved after two weeks in 44.8% of the subjects, and in 71% of them it happened after the third week. Overall, 29% of patients continued viral shedding three weeks after disease onset. The prolongation of viral shedding was higher in hospitalized cases compared to outpatients (*P*value = 0.04). Moreover, low hemoglobin was associated with longer viral shedding.

Viral shedding duration in an infection period must be taken into attention in order to define an appropriate span of isolation which is applied as a marker of infectivity and quarantine guidelines are mostly developed to flash it [9].

It has been demonstrated that viral shedding lasted for up to 63 days after symptoms initiation [9, 27, 28]. To develop the quarantine guidelines, the difference between infectivity and viral shedding is a crucial key.

In a study, viral RNA was determined in COVID-19 infected cases one to three days sooner than symptoms initiation. In addition, viral load reached a peak during the first week in the upper part of the respiratory tract (URT) which then showed a gradual decrease over time. Furthermore, the viral load had a peak during the second week in the lower respiratory tract (LRT) and feces. The severe form of the illness did not affect viral RNA detection although there was an association between longer viral RNA detection and the severe form of the illness [29].

Furthermore, viral RNA detection does not definitely represent the infectiousness of subjects nor their ability in viral transmission to another person. According to the available data, isolation of live viruses is difficultly possible eight to nine days after symptoms start. Nevertheless it could be achieved from respiratory samples until day 18 after symptom presentation which supports the transmission possibility up to the mentioned time [9]. It was reported that viral RNA shedding from the sputum continued even after symptoms clearance. Moreover, seroconversion was tracked in 50% of patients during 7 days, which was not followed by a rapid depletion in viral load. The findings indicate that high viral shedding occurs in the early course of infection when patients present mild symptoms which may explain the cause of the rapid spread of SARS-CoV-2 [18].

Zheng et al. assessed SARS-CoV-2 RNA in various samples. 95% of respiratory track samples were COVID-19 positive during the first 7 days and 54% during the four weeks after symptom onset. The cases who presented the mild form of the infection were infected for an average of 14 days, with a high viral load during the first 2 weeks, whereas the severe cases who experienced shedding for an average of 21 days, with a high viral titration in the second 2 weeks. The associated factors with viral shedding included male gender, age >60 and corticosteroid treatment >10 days which led to longer viral shedding among patients who presented with severe forms of the disease [21].

Yu et al. in a retrospective study on 410 discharged cases showed that 96% of the subjects achieved negative PCR in 30 days. Nearly 9% of the mentioned cases showed resolution of fever, whereas four still had a high temperature. Furthermore, the risk factors associated with viral shedding were explored and revealed that patients with coronary heart disease (CHD) had prolonged viral RNA shedding. Finally, patients with lower albumin levels demonstrated prolonged viral RNA shedding [30].

In the other investigation, COVID-19 cases were followed to achieve a negative PCR. The median viral shedding was obtained at 34.16 days which was not associated with sex, age, or underlying diseases. However, shivers and body pain were seen in the prolonged form of the infection [7].

Qi et al., studied a total of 147 patients with COVID-19 in which 17 days of viral shedding was determined as the median time (varying from 12 to 21). They also observed that the high temperature at the time of admission and hospital length of stay were correlated risk factors to prolonged viral shedding [25].

Liu et al. [20] carried out another research on 21 serial nasopharyngeal swabs obtained from confirmed subjects and found that all the subjects with severe forms of the illness had viral shedding, whereas 90% of patients with mild disease cleared the virus 10 days after symptom onset. In the other study by To et al. on 21 patients, seven subjects had detectable viral load beyond three weeks after symptom onset. Nevertheless, there was no association between prolonged viral shedding and disease severity [9]. The recent data by Glans et al. showed that SARS-CoV2 specific antibodies present in serum may result in a lower risk of SARS-CoV-2 shedding by hospitalized COVID-19 patients [31].

In the study by Chen et al., the investigated patients were categorized into asymptomatic carriers, symptomatic patients, and presymptomatic COVID-19 patients. Asymptomatic subjects included younger individuals with a shorter duration of viral shedding. Viral shedding duration took a longer time in presymptomatic patients in comparison with asymptomatic carriers. They concluded that higher antiviral immunity might be present in asymptomatic carriers of SARS-CoV-2 and that this feature might stem from innate and adaptive cellular immunity. Finally, the severity of the infection was associated with older age [32].

A 71-year-old woman from China was admitted to a hospital with a two-week history of illness [27]. Her documented viral shedding RT-PCR testing of SARS-CoV-2 prolonged for two months after symptoms initiation. According to the report, it was the longest period of viral shedding that had happened to that date, indicating that SARS-CoV-2 viral shedding after COVID-19 diagnosis could be prolonged.

A total of 86 confirmed subjects were studied by Hartman et al., of whom 11 participants were still positive after symptom resolution at a median of 19 days. They reported that older patients had a higher rate of a positive test. They concluded that their results highlighted the testing COVID-19 necessity of convalescent plasma donors less than 28 days after symptom resolution and also suggest that COVID-19 positive patients would be better to be quarantined more than 14 days following recovery recommendation [33].

Finally, the conducted study hereby includes some limitations such as a limited number of samples. Moreover, the PCR test could have been more than three for each patient to determine the viral shedding duration more exactly.

## 5. Conclusion

Almost 30% of patients continued viral shedding after three weeks from disease onset. The longer duration of viral shedding in hospitalized cases rather than outpatients in this study indicates the necessity to design a strategy of isolation for confirmed cases and optimization of interventions. Specifically, a longer duration of isolation in hospitalized patients must be emphasized. The results similar to other investigations call into question if the current policies are enough to prevent the viral spread or not. Furthermore, the study should be carried out on a larger sample size considering the molecular and serological tests assessment in patients even with symptom or without it and also evaluating the virus viability in cell culture.

## Figures and Tables

**Table 1:** Demographic, underlying condition, treatment, chest CT scan findings, and outcomes of COVID-19 patients (*n* = 58).

Data		N (%)
Age	Min	21
	Max	87
	Mean	49.36

Gender	Male	28 (51.7)
	Female	30 (48.3)

Underlying condition	Heart disease	1 (1.7)
	Lung disease	1 (1.7)
	Diabetes	4 [6]
	Immunosuppressive medicine application	1 (1.7)
	Hypertension	2 (3.4)
	Hypertension + heart disease + diabetes	1 (1.7)
	Hypertension + diabetes	1 (1.7)
	Heart disease + diabetes	1 (1.7)

Patients' outcome	In ward	32(55%)
	Outpatients	21(36%)
	ICU	5(9%)
	Oxygen therapy	30(51.7)
	Ventilation	3(5.2)
	Discharge	57
	Death	1

Treatment	Antibiotics	55 (94.8)
	Chloroquine	21 (36.2)
	Chloroquine + kaletra	18 [31]
	Chloroquine + Atazanavir	12 (20.6)
	Chloroquine + kaletra + Ribavirin	5 (8.6)
	Kaletra	2 (3.4)

Chest CT scan	Yes	52 (89.7)
	No	6 [11]
	Normal	8 (15.4)
	Abnormal	44 (84.6)

## Data Availability

All data that support the findings of this study are available from the corresponding author upon reasonable request.

## Abbreviations

SARS-CoV-2: Severe acute respiratory syndrome coronavirus 2
COVID-19: Coronavirus disease 2019
RT-PCR: Real-time PCR.
